# Comparison of PECS I Block and Local Anesthetic Infiltration for Pain Management in Breast Augmentation: A Prospective, Split-Body Study

**DOI:** 10.1007/s00266-026-05885-1

**Published:** 2026-04-30

**Authors:** Mert Ersan, Ozge Koner

**Affiliations:** 1 Department of Plastic, Reconstructive and Aesthetic Surgery, Yeditepe University, Yeditepe Kozyatagi Hospital, Icerenköy Mah., Hastahane Cad. No: 4/1, Atasehir, Istanbul, Turkey; 2 Department of Anesthesiology and Reanimation, Yeditepe University, Istanbul, Turkey

**Keywords:** Augmentation mammoplasty, Breast augmentation, Pain management, PECS, Pectoral nerve block

## Abstract

**Background:**

While various analgesic methods have been proposed in breast augmentation, limited data exist comparing their efficacy in a controlled setting. This prospective, split-body study aimed to compare the effectiveness of preoperative PECS I block and intraoperative direct local anesthetic infiltration for postoperative pain control following breast augmentation.

**Methods:**

Twenty female patients (ASA I–II, aged 18–59) undergoing bilateral breast augmentation with identical implants via dual-plane or submuscular approaches were included. Each patient received a PECS I block on the right breast (10 mL of 0.5% bupivacaine) performed preoperatively by the same anesthesiologist. The left breast received an identical dose via direct local anesthetic infiltration administered by the same plastic surgeon prior to muscle transection. Postoperative pain was evaluated using the Numeric Rating Scale (NRS) at 30 min intervals for the first 2 h, every 2 h between 2 and 6 h, at 24 h, on postoperative day 5, and at 3 months.

**Results:**

Pain scores were significantly lower on the PECS I block side compared to the local infiltration side during the early postoperative period (30 min to 6 h) (*p* < 0.05). No significant difference was observed at 24 h, day 5, or 3 months. The mean tramadol requirement in the first 6 h was 72.0 ± 10.05 mg, with a total of 171.0 ± 21.98 mg at 24 h.

**Conclusions:**

In this split-body study, PECS I block provided superior pain control during the early postoperative period (first 6 h) compared to direct local infiltration.

**Level of Evidence II:**

Evidence Based Medicine Level: Evidence Level II, Therapeutic Study. This journal requires that authors assign a level of evidence to each article. For a full description of these Evidence-Based Medicine ratings, please refer to the Table of Contents or the online Instructions to Authors www.springer.com/00266.

**Supplementary Information:**

The online version contains supplementary material available at 10.1007/s00266-026-05885-1.

## Introduction

In breast augmentation, the surgical approach depends on patient anatomy and desired outcomes, with each technique offering specific considerations [1]. Techniques involving muscle dissection, such as submuscular and dual-plane placements, are associated with greater postoperative pain than subglandular and subfascial methods [2]. Effective pain control is thus essential to optimize recovery and satisfaction [3]. Common strategies include pectoral (PECS) blocks, local anesthetic infiltration, and pocket irrigation [4–6]. The PECS I block is an ultrasound-guided technique targeting the medial and lateral pectoral nerves by injecting anesthetic between the pectoralis major and minor muscles [7].

While multiple studies have investigated the effectiveness of regional anesthesia techniques in breast augmentation, direct comparisons between PECS I blocks performed by anesthesiologists and local anesthetic infiltration administered by plastic surgeons remain limited. This study aims to address this gap by employing a prospective, double-blind, split-body design to directly compare these two techniques in the same patient, thereby eliminating interpatient variability and standardizing the anesthetic agent, volume, and assessment intervals.

## Methods

### Study Design, Inclusion, and Exclusion Criteria

This prospective, double-blind, split-body study was conducted at the Yeditepe University Department of Plastic, Reconstructive and Aesthetic Surgery in collaboration with the Department of Anesthesiology. The study protocol was approved by the Yeditepe University Institutional Ethics Committee (Approval No: 2024-KAEK-21/1031) and registered at ClinicalTrials.gov (NCT06719726).

The same anesthesiologist (O.K.) performed PECS I blocks on the right breasts, while the same plastic surgeon (M.E.) performed all surgeries and local anesthetic infiltrations on the left breasts. Postoperative pain assessments were conducted by a blinded anesthesiologist.

Eligible participants were female patients aged 18–59 years, classified as American Society of Anesthesiologists (ASA) I or II, undergoing primary bilateral breast augmentation with silicone implants using either a dual-plane or submuscular approach, with identical implants on both sides. Exclusion criteria included planned subglandular or subfascial placement, revision surgery, breast disease, prior breast or axillary surgery, or a cancer diagnosis. Patients with preoperative breast pain or chronic pain syndromes (e.g., fibromyalgia, migraines, back pain, complex regional pain syndrome, neuropathic pain, osteoarthritis) were also excluded. Additional criteria were a Breast Imaging Reporting and Data System (BI-RADS) score >3 in the past year, coagulopathy, or current/potential pregnancy.

### Anesthesia & PECS I Block

All procedures were performed under general anesthesia. Induction was achieved with intravenous administration of propofol (2 mg/kg) and fentanyl (2 mcg/kg). A weight-appropriate supraglottic airway device secured the airway. Anesthesia was maintained with sevoflurane (minimum alveolar concentration value: 0.9–1.2) in a 35% oxygen-air mixture and a 0.1–0.4 mcg/kg/min remifentanil infusion. 2 g of intravenous cefazolin was administered for infection prophylaxis.

**Fig. 1:**
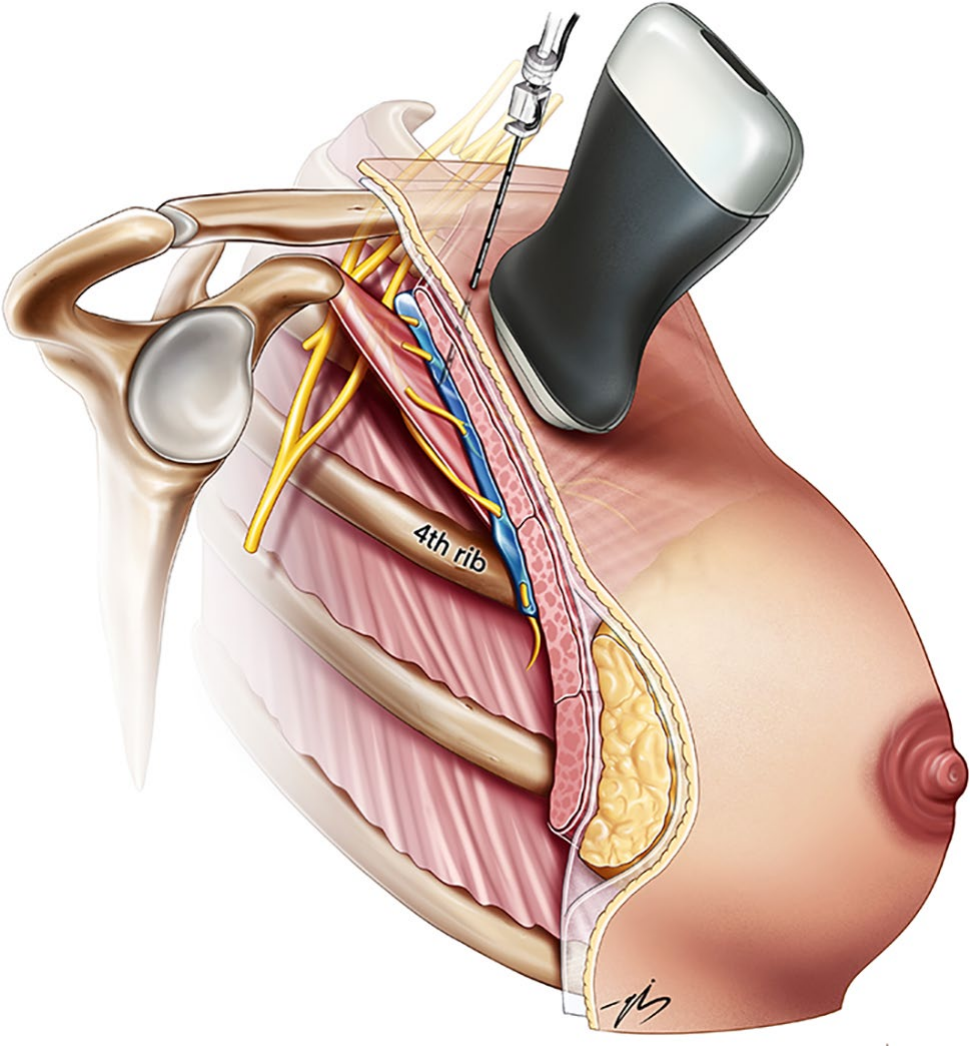
Illustration of PECS I block

**Fig. 2:**
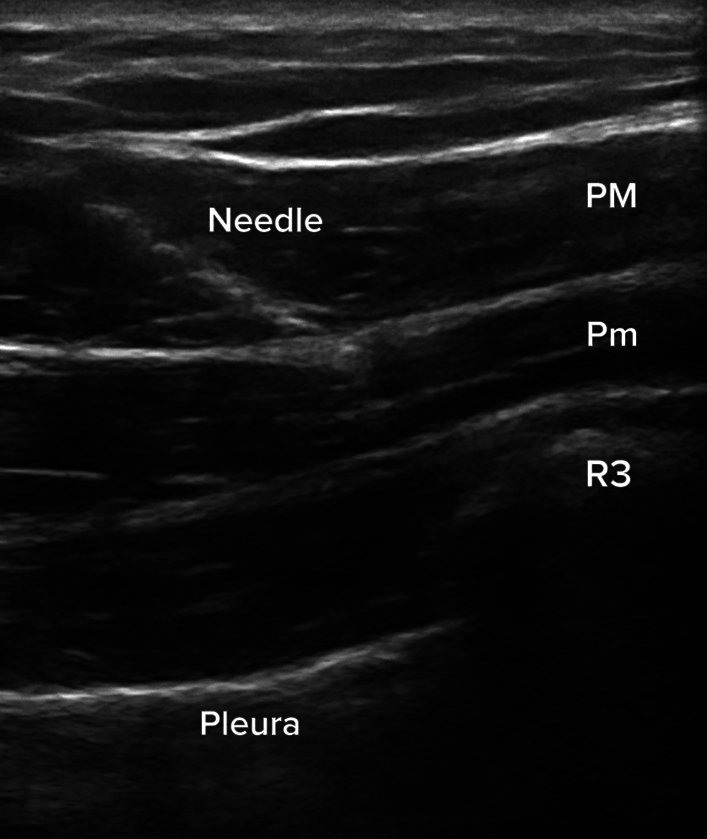
Ultrasound image of PECS I block. The needle is in the interfascial plane between the pectoralis major and pectoralis minor muscles. *PM* Pectoralis major, *Pm* Pectoralis minor, *R3* 3rd rib

Before the surgical procedure, the PECS I block was performed on the right breasts. After disinfection, the patient was placed in a supine position with the arm abducted to 90°. The block was administered using a handheld high-frequency ultrasound device (Clarius L20, Clarius Mobile Health, Canada). The fascia between the major and minor pectoralis muscles and the thoraco-acromial artery's pectoral branch were visualized. In the paramedian sagittal plane, the coracoid process was identified. The ultrasound transducer was then maneuvered proximally and medially to laterally in an in-plane orientation. This allowed for continuous visualization of the nerve block needle (StimuplexR Ultra 360R, 5 cm, BBraun Melsungen, Germany). At the level of the third rib, the needle trajectory and tip were monitored in real time using ultrasound to ensure accurate placement within the targeted fascial plane. To facilitate hydrodissection, 2–3 mL of saline was injected between the pectoral muscles. The final block was administered with 0.2 mL/kg of 0.5% bupivacaine, ensuring adequate regional anesthesia for the surgical procedure. Figure 1 illustrates the PECS I block; Fig. 2 presents the ultrasound image of the PECS I block; and the video demonstrates the application of the PECS I block using a portable high-frequency ultrasound device.

### Surgical Technique and Local Anesthetic Infiltration

Following anesthesia induction and right-sided PECS I block, patients were handed over to the plastic surgeon. All procedures were performed with the patient in the supine position through 4-cm inframammary incisions. The skin and subcutaneous tissues of the left breast were incised, exposing the inferolateral border of the pectoralis major muscle. A solution containing 0.2 mL/kg of 0.5% bupivacaine (same dose as the PECS I block), diluted with 100 mL of sodium chloride, was prepared. This solution was injected into the pectoralis major muscle using a blunt cannula and along the intended cut edges of the muscle under direct vision, as described in previous studies [4, 5, 8, 9].

The procedure was then continued on the right side (PECS I block side). The skin and subcutaneous tissues were incised, and the inferolateral border of the pectoralis major muscle was exposed. In submuscular cases, inferior muscle attachments were transected, and a pocket appropriate for the implant was created. In dual-plane cases, subglandular dissection was performed up to the required level, followed by transection of the inferior muscle attachments as described in the submuscular technique to complete pocket formation. Pocket dissection and release of the pectoralis major muscle were performed using monopolar electrocautery under direct vision. Visible larger vessels were prophylactically coagulated with bipolar electrocautery prior to potential bleeding, and limited blunt dissection was performed when necessary to complete pocket formation.

The surgeon then returned to the left side, where identical steps were repeated for pocket preparation. Implants were placed bilaterally under sterile conditions. The inframammary folds were reinforced using 2/0 polydioxanone sutures. Incisions were closed in layers using 3/0 polydioxanone for the deep layers, 4/0 poliglecaprone for the subdermal layer, and 5/0 poliglecaprone. No drains were used in any patient. The operation was concluded following the application of a medical bra and chest band.

### Postoperative Period and Analgesia Protocol

Postoperatively, all patients received 1 g intravenous paracetamol and a loading dose of 1.5 mg/kg intravenous tramadol. Intravenous dexamethasone (0.1 mg/kg) was administered as antiemetic prophylaxis and as part of multimodal analgesia. Pain control was maintained with a patient-controlled analgesia (PCA) device delivering 20 mg intravenous tramadol (5 mg/mL) every 8 min. If pain persisted (NRS > 3) or the tramadol limit was reached, 1 mg intravenous morphine was planned as rescue analgesia. Patients were instructed to limit bilateral arm movements until discharge to minimize variability in pain perception related to arm use. Patients were assessed for hematoma formation during the hospital stay, and after 24 h they were discharged with oral paracetamol (500 mg, four times daily).

On postoperative day 3, dressings were removed. At the first postoperative week, incision sites were evaluated. At the second postoperative week, chest band was removed and the patients were instructed to begin breast massage. The patients were instructed to wear the medical bra for a duration of one month. Scar management therapy was initiated at the first postoperative month. Subsequent follow-up visits were conducted at postoperative month 3, month 6, and at 1 year. Standardized clinical photographs were obtained at each visit using a digital camera (Canon EOS 5D Mark II, Canon Inc., Tokyo, Japan).

### Evaluation of Postoperative Pain

Patients were blinded to the side of intervention and pain assessments were performed side-specifically without informing the patient which technique was applied to each side. Pain was assessed using the 11-point Numeric Rating Scale (NRS; 0 = no pain, 10 = unbearable pain). Assessments were performed every 30 min for the first 2 h, every 2 h between 2 and 6 h, and at the 24th postoperative hour. The highest pain score between 6 and 24 h was recorded. Pain was not routinely assessed at 12 h due to patients’ sleeping time, but additional analgesics and pain scores were noted if needed. NRS scores were also evaluated on postoperative day 5 and at 3 months for both breasts.

### Statistical Analysis

A power analysis was performed at the beginning of the study to determine the required sample size. The calculation was based on detecting a 20% difference in mean pain scores between the two sides, as assessed by the NRS during the first 6 h, with an alpha level of 0.05 and a beta of 0.90 [10]. Data were analyzed using the Statistical Package for Social Sciences (SPSS 29.0, IBM, NY, USA) and GraphPad Prism (version 9.0, GraphPad, San Diego, CA, USA). NRS scores were reported as mean ± standard deviation (SD) and median (minimum–maximum). The Wilcoxon signed-rank test was employed to compare dependent groups, with *p* < 0.05 considered significant.

## Results

### Demographic, Operative, and Postoperative Data

A total of 20 patients were included in the study, and all participants completed the study without dropout. The mean age was 34 ± 9 years and BMI was 25.2 ± 3.4 kg/m^2^. Mean durations were 90 ± 9 min for surgery, 112 ± 17 min for anesthesia, and 5 ± 0.8 min for PECS I block (Table 1).

**Table 1:** Patient demographics and operative details

	Mean (±SD)
Age (year)	34 ± 9
BMI (kg/m^2^)	25.2 ± 3,4
ASA status, n (%)	20
ASA 1	12 (60%)
ASA 2	8 (40%)
Duration of surgery (mins)	90 ± 9
Duration of anesthesia (mins)	112 ± 17
Duration of PECS 1 block (mins)	5 ± 0,8
Implant volume (cc)	340 ± 26
Plane (n)	20
Dual plane 1	5 (25%)
Dual plane 2	5 (25%)
Dual plane 3	5 (25%)
Submuscular plane	5 (25%)

**Fig. 3:**
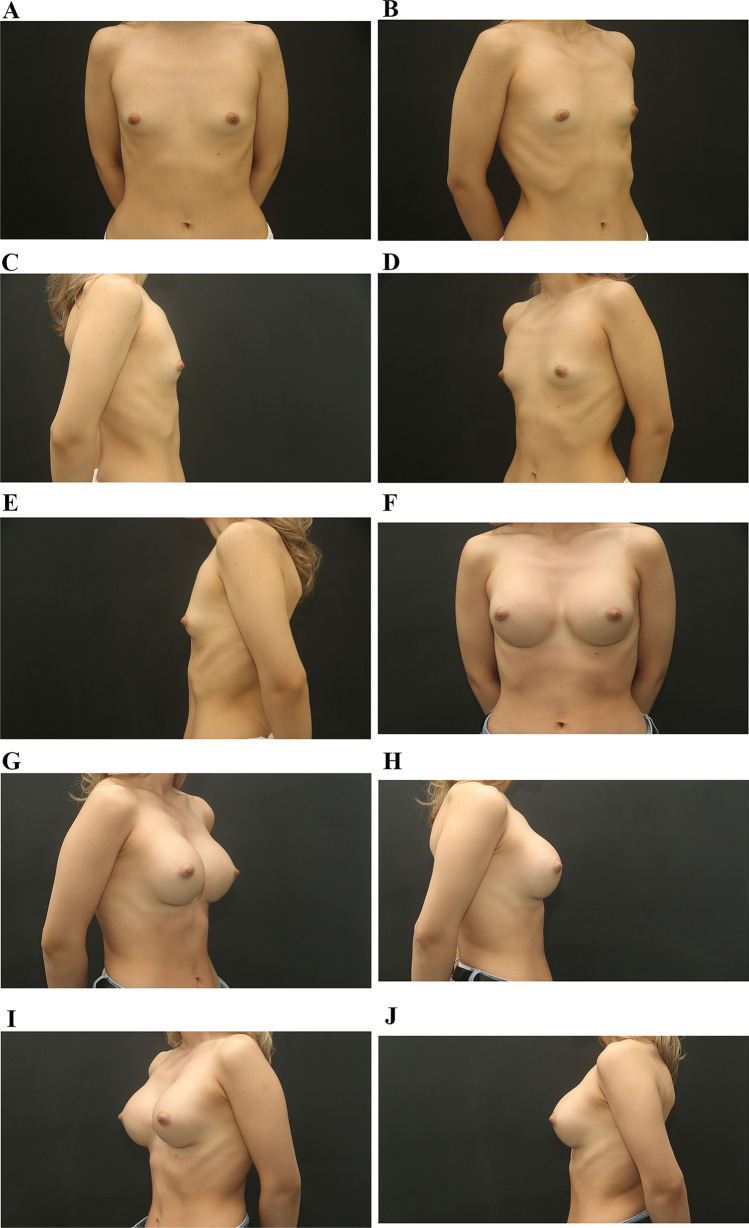
Preoperative and postoperative 1-year views of a 26-year-old patient who presented with hypoplastic breasts and underwent augmentation with a 340 cc Mentor® SILTEX Microtextured Round High Xtra implant placed in a dual-plane 2 pocket. **A** Preoperative frontal view. **B** Preoperative right oblique view. **C** Preoperative right lateral view. **D** Preoperative left oblique view. **E** Preoperative left lateral view. **F** Postoperative 1-year frontal view. **G** Postoperative 1-year right oblique view. **H** Postoperative 1-year right lateral view. **I** Postoperative 1-year left oblique view. **J** Postoperative 1-year left lateral view

All patients received identical implants (Mentor^®^ SILTEX Microtextured Round High Xtra, JNJ MedTech, California, USA) with a mean volume of 340 ± 26 cc (range: 150–455 cc). A dual-plane technique was used in 75% (n = 15), evenly distributed among types I–III. The remaining 25% (n = 5) underwent submuscular placement (Table 1)*.* Figure 3 presents the preoperative and postoperative 1-year images of a 26-year-old patient who presented with hypoplastic breasts and underwent augmentation with a 340 cc Mentor® SILTEX Microtextured Round High Xtra implant placed in a dual-plane 2 pocket.

No anesthesia- or surgery-related complications were noted. There were no cases of capsular contracture, hematoma, infection, rupture, or leakage. Temporary nipple hypoesthesia occurred in 15% (n = 3) and resolved within 3 months. One patient (5%) developed minor wound dehiscence, which healed conservatively.

Mean perioperative remifentanil use was 323 ± 95 mcg. Tramadol use averaged 72.0 ± 10.05 mg in the first 6 h and 171.0 ± 21.98 mg over 24 h (Table 2)*.* No patients experienced opioid-related side effects, such as constipation, respiratory depression, or vomiting; none required intravenous morphine HCl. Nausea developed in 2 patients (10%) and was resolved with appropriate antiemetic treatment.

**Table 2:** Tramadol usage and opioid-related side effects

	Mean (±SD)
Perioperative remifentanil dose (mcg)	323 ± 95
Tramadol requirement in the first 6 h (mg)	72.0 ± 10.05
Tramadol requirement in the first 24 h (mg)	171.0 ± 21,98

### Pain (NRS) Scores

**Fig. 4:**
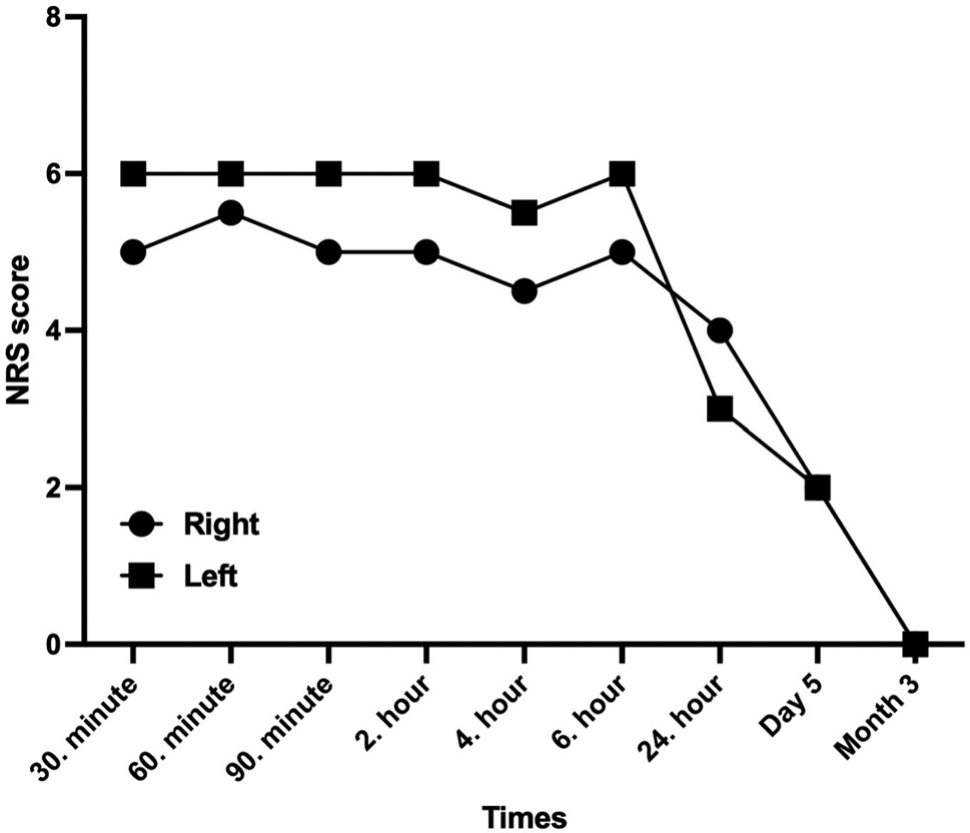
Postoperative pain assessment with the Numeric Rating Scale (NRS) comparing pain in both study groups

Pain intensity on the right side (PECS I block group) was significantly lower than on the left side (local anesthetic infiltration group) at 30 min, 90 min, 2 h, 4 h, and 6 h postoperatively (*p* < 0.05). Mean pain intensity during the first six postoperative hours was consistently higher on the left side than on the right (*p* < 0.05). Beyond the 24 h mark, pain scores in both groups were comparable, with no statistically significant differences observed at the 24th h, 5th postoperative day, or 3rd month (Table 3, Fig. 4).

**Table 3:** Comparison of pain scores between PECS I and local anesthetic infiltration groups

Time	Right breast	Left breast	*p*-value		
	Mean ± SD	Median (Min–Max)	Mean ± SD	Median (Min–Max)	
30 minute	**4.8±1.2**	**5.0 (3.0**–**7.0)**	**5.9±1.1**	**6.0 (4.0**–**8.0)**	**0.025**
60 minute	5.2±1.5	5.5 (3.0–7.0)	6.3±1.1	6.0 (4.0–8.0)	0.054
90 minute	**5.1±1.2**	**5.0 (3.0**–**7.0)**	**5.9±1.4**	**6.0 (4.0**–**8.0)**	**0.023**
2 hour	**5.1±1.2**	**5.0 (3.0**–**7.0)**	**6.0±1.2**	**6.0 (4.0**–**8.0)**	**0.027**
4 hour	**4.7±1.3**	**4.5 (3.0**–**7.0)**	**5.4±1.2**	**5.5 (3.0**–**8.0)**	**0.044**
6 hour	**4.7±1.3**	**5.0 (3.0**–**7.0)**	**5.8±1.2**	**6.0 (4.0**–**8.0)**	**0.040**
24 hour	4.0±0.9	4.0 (2.0–5.0)	3.5±1.1	3.0 (2.0–5.0)	0.085
Day 5	2.0±0.7	2.0 (1.0–3.0)	2.1±0.4	2.0 (1.0–3.0)	0.527
Month 3	0.0±0.0	0.0 (0.0–0.0)	0.0±0.0	0.0 (0.0–0.0)	1.000

Bold values indicate statistically significant differences between groups (*p* < 0.05)

## Discussion

Breast augmentation is a common aesthetic procedure to enhance breast volume and shape [11]. Subglandular implants lie beneath breast tissue, while subfascial placement adds fascia coverage [1–3]. Submuscular and dual-plane approaches, preferred for patients with thin tissue, require more dissection and result in greater postoperative pain [2, 12].

Postoperative pain mainly results from disruption and stretching of the pectoralis major muscle and fascia. Dissection may include rib detachment and muscle fiber splitting to access the interpectoral plane, causing myofascial pain mediated by the medial and lateral pectoral nerves. Trauma or tension to the lateral cutaneous branches of the intercostal nerves during implant placement may further increase discomfort [13].

The surgical approach may also influence postoperative pain. Although dual-plane and submuscular techniques involve different extents of tissue dissection, the inferior release of the pectoralis major muscle is performed at a similar anatomical location in both techniques. In dual-plane augmentation, additional subglandular dissection is performed prior to muscle release, whereas submuscular augmentation involves predominantly subpectoral dissection. In the present split-body study, however, the same surgical technique and identical implants were used bilaterally in each patient, thereby minimizing the influence of surgical approach on the comparison between analgesic techniques.

The method of surgical dissection may also influence postoperative pain. Electrocautery-based dissection allows precise tissue division and effective hemostasis, facilitating controlled release of the pectoralis major muscle. However, thermal spread may theoretically contribute to tissue irritation. In contrast, blunt dissection avoids thermal injury but may result in greater mechanical tissue traction. Although these factors could potentially influence postoperative discomfort, the dissection technique was standardized across all patients in the present study, thereby minimizing variability related to the dissection method.

In 2011, Blanco introduced the PECS block as an ultrasound-guided technique involving local anesthetic injection between the pectoralis major and minor muscles to block the medial and lateral pectoral nerves [14]. This approach, termed PECS I, was later expanded into PECS II, which includes an additional injection between the pectoralis minor and serratus anterior to block the long thoracic and lateral intercostal nerves (T3–T6), offering broader analgesia [7]. PECS blocks are now widely used in breast surgeries, including mastectomies [15–25].

Multiple studies have shown that PECS blocks reduce postoperative pain in aesthetic breast augmentation [10, 26–29]. A meta-analysis by Yang et al. confirmed their analgesic efficacy [30]. Duarte et al. reported significant reductions in both pain scores and opioid use [9]. Ekinci et al. found that 20 mL and 30 mL of 0.25% bupivacaine both effectively reduced pain and fentanyl consumption without significant difference [31]. Ciftci et al. also noted significantly lower pain scores and need for rescue analgesia with preoperative PECS I blocks [11].

Beyond ultrasound-guided methods, nerve blocks under direct vision have also shown efficacy. Zhang et al. reported significantly lower pain scores up to 48 h postoperatively using direct-vision PECS blocks during endoscopic-assisted transaxillary augmentation [32]. Similarly, Lee et al. found that interpectoral and subserratus blocks under direct vision reduced opioid use and pain scores in subpectoral augmentations [33]. Williams et al. described a field block technique targeting the intercostal neurovascular bundle, eliminating postoperative opioid prescriptions over four years [34].

Besides nerve blocks, local anesthetic infiltration and pocket irrigation are commonly used for postoperative analgesia due to their simplicity [35]. Jabs et al. found that intraoperative tumescent infiltration with bupivacaine significantly reduced recovery room pain scores and postoperative opioid use by over 50% [8]. Durán-Vega et al. showed that ropivacaine irrigation lowered pain scores at 90 and 120 min postoperatively, though the effect was transient [6]. Similarly, Chen et al. reported short-term pain relief at 1 h using bupivacaine and ketorolac, with no lasting benefit beyond that [35]. These findings suggest that while local irrigation provides temporary relief, it lacks the sustained analgesia achieved by regional blocks like PECS I, which showed superior control up to 6 h postoperatively in our study.

Few studies have directly compared PECS blocks and local infiltration [15]. Lindsey et al. found that ultrasound-guided nerve blocks reduced opioid use and PACU stay compared to blind infiltration in submuscular augmentation [36]. Hakim et al. also reported lower pain scores, delayed need for analgesia, and reduced morphine use with PECS blocks [37]. Heffern et al. showed a 33% drop in PACU opioid consumption and a 13% shorter stay, though discharge pain and operative time were similar [38]. Interestingly, Moris et al. found lower 24 h NRS scores in the infiltration group despite similar scores at earlier time points [4].

Most previous comparisons between PECS blocks and local anesthetic infiltration have relied on separate patient cohorts, introducing potential bias related to interindividual differences in pain perception, anatomical variability, and pharmacologic response. The relatively high standard deviations reported in postoperative pain scores in these studies further support this concern [10, 37]. To address this limitation, the present study employed a prospective, double-blind, split-body design, allowing both analgesic techniques to be evaluated within the same patient under identical surgical and anesthetic conditions. All interventions were performed by the same anesthesiologist and surgeon using identical volumes of 0.5% bupivacaine, and postoperative pain was assessed at predefined intervals. By minimizing interpatient variability, this methodological approach enabled a more controlled comparison of the two techniques and yielded more consistent pain score distributions, with standard deviations remaining approximately 25% of the mean (Table 3).

Our findings showed that PECS I block provided significantly better analgesia within the first 6 h postoperatively compared to local infiltration. No significant differences were observed at 24 h, postoperative day 5, or at the 3-month follow-up. This suggests that PECS I block offers superior short-term pain control, likely due to its targeted blockade of the medial and lateral pectoral nerves, which play a central role in early postoperative myofascial pain following pectoralis major manipulation.

Although the difference in NRS scores between the two techniques reached statistical significance during the early postoperative period, the absolute difference in pain scores was relatively modest. In the context of pain research, a reduction of approximately one point on the Numeric Rating Scale has often been considered a threshold for the minimal clinically important difference (MCID) [39]. Therefore, while the observed reduction in pain scores favors PECS I block, the clinical relevance of this difference should be interpreted within the context of early postoperative pain following submuscular or dual-plane breast augmentation.

PECS I was selected over PECS II to isolate the effect of blocking only the medial and lateral pectoral nerves, the main sources of pain from pectoralis major dissection in submuscular and dual-plane augmentations. Although PECS II provides broader analgesia by also targeting intercostal, thoracodorsal, and long thoracic nerves, its use would have introduced additional variables related to somatic and dermatomal pain [7]. To focus on early myofascial pain and ensure a clean comparison with local infiltration, PECS II was not applied.

There is no consensus on the optimal agent or concentration for PECS blocks in breast augmentation; studies have used 10 mL of ropivacaine (3.75 mg/mL) and 10–30 mL of 0.25% bupivacaine [10, 31, 37]. Although our study used a higher concentration, direct comparison of pain scores is not possible due to the split-body design.

We used tramadol instead of morphine due to its shorter half-life and more favorable side effect profile (Table 2) [40, 41]. Although morphine and fentanyl have also been studied, available data are insufficient for direct efficacy comparisons [42]. Additionally, our split-body design prevented direct comparison of opioid consumption with existing studies. Nausea occurred in only 10% of patients (n = 2), likely due to perioperative tramadol use and dexamethasone administration [43–46].

This is the first prospective study to directly compare PECS I block and local infiltration using a split-body design in breast augmentation. By eliminating interpatient variability, this model enhances the clinical relevance of our findings. Both techniques were standardized in volume and drug type, and pain was assessed by a blinded evaluator. These methodological strengths support the reliability of our results. When early postoperative pain is the main concern, PECS I block appears to be a practical and effective option. To our knowledge, this is also the first study to compare anesthesiologist-performed PECS I blocks with plastic surgeon-administered local infiltration in a standardized, split-body model [15].

The surgical sequence was designed to allow both anesthetic techniques sufficient time to take effect. A PECS I block was performed preoperatively on the right, followed by local infiltration into the left pectoralis major before muscle transection. Pocket dissection began on the right, then continued on the left. This sequence ensured adequate analgesic onset before tissue manipulation on both sides.

Importantly, on the local anesthetic infiltration side, the skin and subcutaneous tissue were incised to expose the pectoralis major muscle, and the anesthetic solution was injected directly into the muscle [4, 5, 8]. This technique targeted the primary source of postoperative pain in breast augmentation—pectoral muscle dissection—unlike blind tumescent infiltration or pocket irrigation techniques, which are typically performed between the breast tissue and muscle or into the pocket without direct visualization of the muscle.

Although infiltration of local anesthetic after muscle transection would have provided easier access to the interfascial planes and allowed direct targeting of the medial and lateral pectoral nerves, this approach would have permitted nociceptive signaling to occur prior to anesthetic action. Such a delay in analgesic onset would have compromised the comparability with the preemptive PECS I block, which was administered before any tissue manipulation. To ensure a valid and standardized comparison between techniques, local anesthetic infiltration was deliberately performed before muscle transection, targeting the pectoralis major muscle under direct vision.

Despite its robust and controlled design, this study has limitations. The sample size, though statistically powered, may not reflect the full range of anatomical variation. Another limitation of this study is that broader patient-reported outcome measures (PROM) evaluating overall patient satisfaction or quality of recovery were not assessed. Although postoperative pain was evaluated using the Numeric Rating Scale, the inclusion of validated PROM instruments in future studies may provide a more comprehensive assessment of patient experience following breast augmentation surgery. Side-specific tramadol consumption could not be evaluated because postoperative analgesia was administered systemically through patient-controlled analgesia (PCA), affecting both breasts equally. Therefore, although PECS I block was associated with lower early postoperative pain scores, the present study design does not allow a definitive assessment of a side-specific opioid-sparing effect. The optimal anesthetic volume and concentration for PECS blocks remain unclear, as studies vary in agents, pharmacodynamics, and dosing protocols. This inconsistency highlights the need for future standardization. To ensure procedural consistency, PECS I was always applied to the right and local infiltration to the left. Although assessments were blinded and patients unaware of sides, fixed lateralization may introduce bias. Randomized side allocation in future studies could reduce this. Another potential source of bias relates to arm dominance. In the present study, the anesthesiologist, the operating surgeon, and all patients were right-hand dominant, and procedures were performed using the dominant hand in a consistent manner on both sides. Although this likely reduced ergonomic variability during the intervention, preferential use of the dominant arm during the early postoperative period could theoretically influence pain perception and should be considered when interpreting the results. All surgeries used an inframammary approach, but other techniques—transaxillary, periareolar, transumbilical—may involve different dissection planes and nerve pathways. Additional studies are needed to confirm whether our results apply to those settings. To improve generalizability, future research should involve larger samples, randomized lateralization, longer follow-up, and comparisons across different anesthesia techniques and analgesics.

## Conclusion

In this split-body study, PECS I block improved pain control during the early postoperative period. Given its ability to enhance early postoperative comfort without increasing complication rates, PECS I block may serve as a valuable component of multimodal analgesia protocols in breast augmentation, particularly for procedures involving muscle dissection.

## Supplementary Information

Below is the link to the electronic supplementary material.

Supplementary file1 (MP4 97003 KB). The application of the PECS I block using a handheld high-frequency ultrasound device.
